# Body Mass Index Impacts on Gray Matter Volume in Developmental Restrictive Anorexia Nervosa: A Voxel-Based Morphometry Study

**DOI:** 10.3390/nu17162620

**Published:** 2025-08-13

**Authors:** Olivia Curzio, Carlotta Francesca De Pasquale, Sandra Maestro, Vittorio Belmonti, Laura Biagi, Michela Tosetti, Filippo Muratori, Rosa Pasquariello, Alessandra Retico, Sara Calderoni

**Affiliations:** 1 Institute of Clinical Physiology, National Research Council, Via Moruzzi 1, 56124 Pisa, Italy; olivia.curzio@cnr.it; 2 Cantonal Sociopsychiatric Organisation, Public Health Division, Department of Health and Social Care, Republic and Canton of Ticino, 6850 Mendrisio, Switzerland; carlotta.depasquale@eoc.ch; 3 Child and Adolescent Rehabilitation Clinic “Gli Orti di ADA”, Via dei Giacinti, 4, 56128 Pisa, Italy; sandra.maestro@hotmail.com; 4 IRCCS Stella Maris Foundation, Viale del Tirreno 331, 56128 Pisa, Italy; vittorio.belmonti@fsm.unipi.it (V.B.); laura.biagi@fsm.unipi.it (L.B.); michela.tosetti@fsm.unipi.it (M.T.); rosa.pasquariello@fsm.unipi.it (R.P.); 5 Stella Maris Mediterraneo Foundation, 85032 Chiaromonte, Italy; filippo.muratori@fsm.unipi.it; 6 Pisa Division, National Institute of Nuclear Physics, Largo Bruno Pontecorvo 3, 56127 Pisa, Italy; alessandra.retico@pi.infn.it; 7 Department of Clinical and Experimental Medicine, University of Pisa, 56126 Pisa, Italy

**Keywords:** anorexia nervosa restricting type, adolescence, brain volume, voxel-based morphometry, structural magnetic resonance, eating disorders, body mass index, T1-weighted MRI

## Abstract

**Background/Objectives:** Previous magnetic resonance imaging (MRI) investigations reported brain alterations in anorexia nervosa restricting type (AN-R); however, the number of existing structural neuroimaging studies in the developmental age is limited. Here, we analyzed the volumetric brain differences between adolescent patients with AN-R and control peers, and possible correlations between brain volumes and clinical features. **Methods:** The sample comprised 47 adolescent females with AN-R (mean age: 15.0 years, SD = 1.4) who underwent structural MRI within one month of admission to a tertiary care university hospital, and 39 typically developing controls matched for sex and age. The patients were clinically characterized by standardized interviews/questionnaires. Using the voxel-based morphometry (VBM) technique, possible significant volumetric brain differences between the two groups were analyzed. Moreover, correlations between altered brain regions and clinical (i.e., body mass index (BMI) and disease duration) or psychopathological variables were investigated. **Results:** An overall reduction in gray matter (GM) volume with a concomitant increase in cerebrospinal fluid (CSF) is observed in AN-R patients; these alterations correlate with a lower BMI. The reduction in GM volume affects the frontal and parietal regions involved in the cognitive processes that underlie and sustain the AN-R clinical features. **Conclusions:** These results add to the current knowledge of the AN-R pathophysiology and pave the way for the development of brain imaging biomarkers for AN in the developmental age.

## 1. Introduction

Previous studies have indicated that the brain volume is typically reduced in acute anorexia nervosa (AN), with one of the largest effect sizes among psychiatric disorders [1]. Brain MRI studies in AN patients showed consistent findings of sulcal widening, ventricular enlargement, and reduced gray and white matter volumes (GM and WM), although the relationship between these changes and clinical features (e.g., weight and illness duration) remains controversial [2]. Key cerebral structures altered in AN include the frontal lobes (implicated in executive function deficits), the parietal cortex (linked to body image distortions), the amygdala (related to anxiety responses), and the striatum (connected to obsessive-compulsive features). In particular, the orbitofrontal cortex (OFC) is involved in processing satiety signals to regulate food intake [3,4]. In addition, the alteration in the insula, a region crucial for taste and interoceptive awareness that integrates external and internal signals, may play a critical role in the AN pathophysiology [5,6]. Brain imaging studies on these regions have yielded mixed results, with increased, decreased, or unchanged brain volumes in AN compared to healthy controls [2,7,8]. Decreased GM volumes in the frontal lobes have been documented in AN, starting with a whole brain voxel-based morphometry study [9], and confirmed in subsequent investigations [10,11]. Structural changes in regions associated with cognitive and emotional processing, such as the anterior cingulate cortex (ACC) and the supplementary motor area (SMA), may underlie some key features of AN psychopathology, including cognitive rigidity and perfectionism [12,13]. Joos et al. [14] also found similar changes in the ACC and frontal operculum. A reduced ACC volume has been reported in adolescents with restricting-type AN (AN-R) in the early stages of the disease [15,16]. Other studies have identified reduced GM in AN patients involving widespread brain regions [17], or the right hippocampus, left middle, and right inferior frontal gyri [18].

More recently, a large MRI study found that early-onset anorexia nervosa (<13 years) was characterized by widespread cortex thinning [19], whereas an investigation on adolescent girls with first-onset AN detected a significant decrease in total and cortical gray matter volumes, especially in the frontal and parietal cortices [20]. A whole-brain analysis by our group [21] confirmed the findings of reduced gray matter and increased cerebrospinal fluid in AN-R, with regional differences observed in the frontal lobes and left insula. These areas are associated with emotional and cognitive deficits in AN, such as difficulties in reward processing and decision-making. A systematic review also noted significant reductions in the global GM volume in AN patients compared to controls [22]. Kohmura et al. [23] found decreased GM in regions such as the superior temporal gyrus (linked to body processing), middle temporal gyrus (related to facial recognition), and pulvinar (implicated in visual processing), after correcting for BMI and age. Most of these findings are based on regional volume comparisons, whereas voxel-based morphometry (VBM) offers a more comprehensive approach by analyzing voxel-level volumetric differences across the entire brain, avoiding the limitations of the region-specific analyses [24].

The aim of the present study was to evaluate brain volume on a voxel-by-voxel basis (i.e., VBM) to detect any differences between adolescents with AN-R and healthy controls, accounting for clinical, demographic, and psychopathological characteristics.

## 2. Materials and Methods

### 2.1. Participants

Clinical records of 124 patients consecutively referred from May 2013 to May 2019 to the Feeding and Eating Disorders Unit of the IRCCS Fondazione Stella Maris (Pisa, Italy) have been examined. All female patients with a clinical diagnosis of AN-R and who had performed structural magnetic resonance imaging (MRI), as per the current clinical protocol, were enrolled. The exclusion criteria were as follows: male sex; neurological syndromes or focal neurological signs; genetic syndromes; and structural anomalies to MRI. According to a neuroradiological evaluation, non-specific alterations, such as minimal dilations of the perivascular spaces, slight asymmetries of the lateral ventricles, or small arachnoid cysts, were not considered significant for exclusion from the study. The final sample consists of 47 patients with AN-R and 39 healthy controls, within an age range of 10–19 years. The control group was selected from a database of subjects who underwent MRI at our institute for various reasons, including headaches, syncopal episodes, or research purposes.

### 2.2. Clinical and Psychopathological Assessment

The body mass index (BMI) at the time of the image acquisition was calculated from daily weight monitoring with the height as measured at the hospital admission. The duration of illness was calculated as the time elapsed from the onset of eating disorder symptoms until the moment of acquisition of the neuroimaging. All patients were following a dietary meal plan agreed with the clinical nutritionist and pediatrician. The presence of amenorrhea and hyperactivity was obtained from anamnestic data and from a pediatric specialist evaluation.

All patients underwent a psychopathological assessment, consisting of clinical interviews and standardized questionnaires (current clinical protocol). Informed consent to data processing was acquired from parents or legal tutor of the minors. An AN-R diagnosis was made after administration of the semi-structured K-SADS-PL clinical interview with both patients and parents [25]. Patients diagnosed before 2013 met the criteria for AN-R according to the DSM-IV-TR criteria. These diagnoses were subsequently re-examined considering the DSM-5 diagnostic criteria, and all patients were confirmed as affected by AN-R, satisfying the DSM-5 criteria, so that they could be homologated to the diagnoses issued for patients after 2013. The presence of a dysfunctional personality organization, investigated through targeted clinical interviews, was verified through a SCID-II screening questionnaire [26], {Citation}which allows for a categorical assessment of personality disorders according to DSM. All the mothers of the patients filled in the Child Behavior Checklist 6/18 questionnaire (CBCL) [27] referring to their daughter. The “Affective Problems” scale of the CBCL, which includes Dysthymia and Major Depression, has been used to have a dimensional measure of mood problems and used as a covariate in statistical analysis. Patients completed the specific Eating Disorder Inventory-3 (EDI-3) questionnaire for Eating Behavior Disorders [28]. The EDI-3 questionnaire consists of 91 items organized in 12 primary scales: drive for thinness, bulimia, body dissatisfaction, low self-esteem, personal alienation, interpersonal insecurity, interpersonal alienation, interceptive deficits, emotional dysregulation, perfectionism, asceticism, and fear of maturity. Six composite scales are obtained from the 12 primary scales; 1 is specific for eating disorders and 5 are general integrative psychological constructs (ineffectiveness, interpersonal problems, emotional problems, excessive control, and general psychological maladjustment) [28]. All patients also completed the Body Uneasiness Test (BUT) [29]. The BUT is composed of 2 parts, 1 first with 34 clinical items and a second with 37 parts, body features, and functions (e.g., stature, nose, body odor) to which the subject could give a discomfort value, which will then come inserted in a rating scale. The BUT returns the following scales: global severity index, fear of weight gain, concerns about the image of one’s body, avoidance behaviors, compulsive checks of one’s body image, and depersonalization. The presence of clinical perfectionism has been studied with the Children and Adolescent Perfectionism Scale (CAPS) [30], consisting of 22 items that evaluate self-oriented perfectionism (SOP) and socially prescribed perfectionism (SPP).

### 2.3. Structural Magnetic Resonance Imaging

All patients underwent MRI scans within a month of entering the ward. MRI data were acquired using a Signa GE 1.5 T Neuro-optimized (GE Healthcare, Milwaukee, WI, USA) system equipped with 40 mT/m high speed gradients. The standard MRI protocol for children and adolescents included several sequences, such as a 2D T1-weighted Spin Echo (SE), a 2D T2-weighted Fast Spin Echo (FSE), a 3D T2-weighted Fluid-attenuated inversion recover (FLAIR), a 3D T2*-weighted Susceptibility-Weighted Angiography (SWAN), and a 2D Diffusion-Weighted Imaging (DWI) acquisition. A whole brain 3D T1-weighted fast-spoiled gradient-recalled echo (FSPGR) imaging sequence included in the protocol as well, providing high-resolution brain images with isotropic voxels of 1 × 1 × 1 mm^3^, was used for structural brain morphometric analysis. The complete protocol lasted about 50 min, while the 3D T1-weighted sequence lasted approximately 8 min.

### 2.4. Statistical Analysis

A VBM analysis was conducted on the T1-weighted MRI images of cases and controls by means of the Statistical Parametric Mapping (SPM) software package (http://www.fil.ion.ucl.ac.uk/spm (accessed on 11 August 2025)), version SPM8 (Welcome Department of Imaging Neuroscience, London, UK). The VBM analysis involves a voxel-wise comparison of tissue volume between groups of subjects using statistical tests. The steps of the analysis included the following: pre-processing of brain images and segmentation of GM, WM, and CSF [31]; implementation of the diffeomorphic anatomical registration using the Lie exponential algebra algorithm (DARTEL) to obtain a population-based brain template [32]; transposition of the DARTEL model in the reference space of the Montreal Neurological Institute (MNI) and consequent diffeomorphic warping of segmented brain tissues in the MNI space; standard smoothing with an isotropic Gaussian kernel (s = 6 mm). The final step of the VBM analysis consists of the statistical comparison between the two groups of subjects according to the general linear model (GLM), which models the effect of variables like group membership or clinical parameters and provides statistical maps that identify statistically significant differences in GM, WM, and CSF volumes.

The two-sample *t*-test was implemented in our analysis to carry out the two-group comparison. Age and TIV were taken into consideration as covariates in the statistical analysis. To avoid possible effects of overlap at the border between the different tissues (edge effects), an absolute threshold of 0.3 was used on the GM, the WM, and the CSF-modulated density maps, thus allowing for a minimal overlap between the segmented brain tissues. A threshold of 500 voxels was set to identify the main regional GM differences between the two groups.

Secondarily, we explored the presence of possible correlations between the areas with a significant between-group difference and the clinical variables (i.e., BMI, disease duration, psychopathological features).

Correlations with brain volumes were searched for the two dimensions of perfectionism evaluated by the SOP and SPP scales of the CAPS questionnaire, and the following EDI-3 subscales have been examined: drive for thinness (DT), body dissatisfaction (BD), risk score for an eating disorder (ERDC), interoceptive deficit (ID); among the composite psychological scales of EDI-3, we assessed the following: hyper-control (OC), inadequacy (IC), affective problems (APCs) and interpersonal problems (IPCs), and the general psychological maladjustment composite (GPMC).

To investigate the aspects related to self and body image, we examined the following scales of the BUT questionnaire: weight phobia (WP), body image concern (BIC), compulsive self-monitoring (CSM), avoidance (A) and depersonalization (D), and the global severity index (GSI). Finally, to evaluate possible correlations with depressive symptoms, we took into consideration the DSM-oriented scale relating to affective problems (AFPs) of the CBCL.3.

## 3. Results

### 3.1. Population

From the original sample of 124 patients, subjects who did not meet the DSM-5 criteria for the clinical diagnosis of AN restrictive subtype were excluded. Specifically, 26 patients were diagnosed with anorexia nervosa binge-purging subtype, 5 with bulimia nervosa, 1 patient had a binge eating disorder, and 8 had other psychiatric diagnoses. In addition, 11 patients were excluded from the study because they were male, and 23 were excluded since they had not performed a structural MRI at our institute or because of the poor image quality, which was insufficient for conducting a VBM analysis. From this pool of 50 patients, 3 other patients were excluded due to subtle structural brain abnormalities on MRI.

### 3.2. Demographic and Clinical Characteristics of the AN-R Study Population

The mean age of the 47 patients with anorexia nervosa restrictive subtype (AN-R) was 14.9 years, with a standard deviation (SD) of 1.4 years, and a range between 10.9 and 18.3 years. The average BMI in the AN-R group was 15.3 kg/m^2^ (SD 1.3). The average age of onset is 13.7 years (SD 1.5), with a range between 10.3 and 16.6 years. The average duration of illness, calculated as the duration from the onset of symptoms to the time of acquisition of the MRI data, is 17 months (SD 9.6), with a range between 2 months and 5 years. Hyperactivity is present in more than half of the clinical population. Amenorrhea was present in 72% of cases in a secondary form, with an average duration of amenorrhea of 7.7 months (SD 3.4). In addition, 13% of patients had primary amenorrhea, and 15% did not have amenorrhea. One patient had polycystic ovary syndrome, not on estrogenic–progestogen therapy (Table 1).

### 3.3. Comorbidity

Based on the CBCL, the average of the scores obtained in the subscale “Affective problems” (AFPs), which includes dysthymic and major depressive symptoms, was 66.6 (SD 8.5). This symptomatology was present in 58% of the sample, equally distributed between symptoms attributable to dysthymia (29%) and symptoms attributable to depression (29%). Within our clinical sample, an anxiety disorder was present in a full-blown manner in one third of the patients, 42% presented anxious traits, and the remaining had no anxious symptoms. Regarding the obsessive-compulsive dimension, a full-blown obsessive-compulsive disorder (OCD) was present in 7% of the sample; however, in about one third of cases, obsessive traits were present. Twenty-six patients were not taking drug therapy at the time of MR imaging. The rest of the sample (n = 21) were taking one or more drugs in the classes of antidepressant, anxiolytic, mood stabilizer, and antipsychotic aimed to support the relative above-mentioned traits, in combination with AN-R diagnosis.

### 3.4. Demographic Data and Global Volume Comparisons

The average age of the control population was 15.3 years (SD 1.96), whereas the age range was 10.6–20 years. This does not statistically significantly differ from the average age of the AN-R population of 14.9 years (SD 1.43), with a range of 10.9–18.3 years. No significant differences were found between the group means at the level of TIV, calculated as the sum of GM, WM, and CSF. Statistically significant differences emerged between the two samples as regards the GM volumes, which were found to be significantly lower in the AN-R subjects compared to the controls, and for the CSF, that was significantly greater in the AN-R subjects compared to controls. No differences were detected for the WM level (Table 2). The differences found between the two populations in terms of GM and CSF were confirmed to be significant (*p* < 0.05), even when age and TIV were considered as covariates in the statistical analysis.

### 3.5. Analysis of Regional Gray Matter Differences

**Figure 1:**
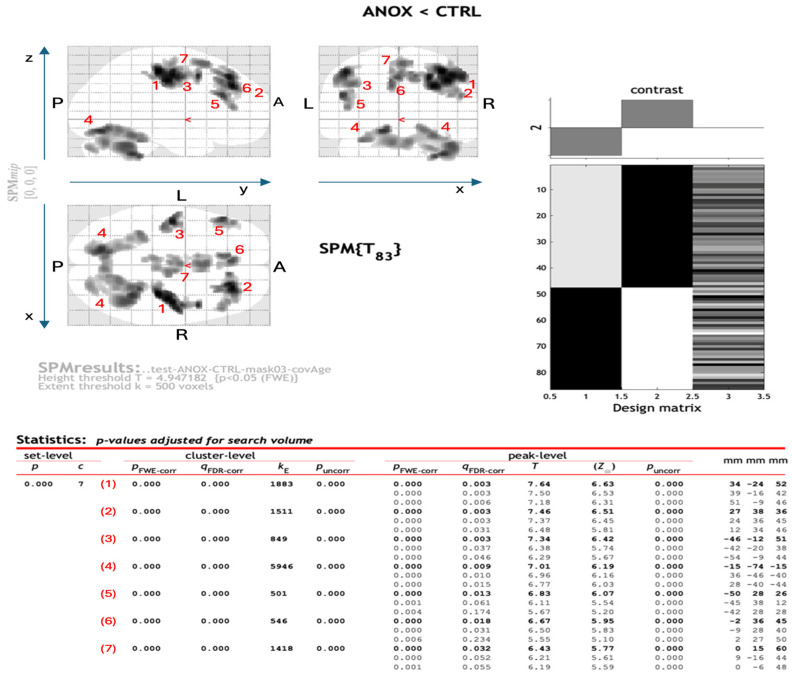
The seven main regions of reduced GM volume in AN-R patients with respect to controls, as reported in the SPM output. The MNI coordinates identify each cluster and sub cluster of voxels where statistically significant volume differences were found. The seven main clusters are labeled with red numbers, which are positioned on the SPM glass brain views (i.e., the maximum intensity projections of significant areas along the x, y, and z orthogonal directions) to allow the identification of the brain areas involved. Further details on the region labels are provided in Table 3.

Figure 1 and Table 3 showed the main GM areas of AN-R patients that significantly differ from the control subjects, obtained by the VBM analysis with a value of *p* < 0.05 and a threshold of 500 voxels to the extent of the detected clusters. Statistically significant differences, in terms of the decrease in the gray matter in the AN-R case compared to the controls, were found in seven main areas, as shown in Figure 1.

Table S1 of the Supplementary Materials reports the results obtained when releasing the constraint on the cluster extent. Several more areas are visible in that case, including the insula.

### 3.6. Correlations Between Neuroimaging Results and Clinical and Psychopathological Data

**Figure 2:**
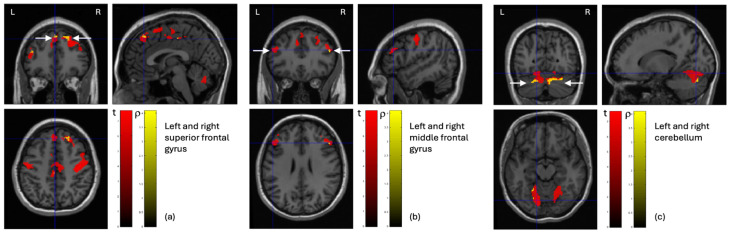
Correlation between regions with decreased GM volume (red voxels show the t maps values obtained for brain regions described in Figure 1 and Table 3) and BMI. The yellow voxels report the maps of the Person correlation between the GM volume at the voxel level and the BMI. Significant correlations were detected for the following GM areas: (**a**) left and right superior frontal gyrus; (**b**) left and right middle frontal gyrus; (**c**) left and right cerebellum.

Possible correlations between the locally altered GM volumes and both clinical dimensions (BMI and disease duration) and psychopathological dimensions (CAPS, BUT, and some subscales of the EDI-3 questionnaire) were investigated in AN-R patients. The analysis was restricted to the GM regions where statistically significant smaller GM volumes were detected between AN-R and control subjects (i.e., those described in Figure 1 and Table 3). No statistically significant correlations emerged, except for the BMI. Figure 2 shows the brain areas where significant Pearson correlation values were found. In particular, whereas the red color scale used for the t value map (obtained with the two-sample *t* test described in the previous section) allows to visualize those voxels with a decreased GM volume in AN-R with respect to controls in Figure 2, the yellow color scale is used to represent the map of the Person correlation values between the GM values in those voxels and the BMI. In both cases, only the statistically significant voxels for the statistical analysis (i.e., the two-sample *t* test and the Person correlation, respectively) are shown.

### 3.7. Psychopathological Dimensions

As for clinical perfectionism, assessed with the CAPS questionnaire, the average of the scores obtained on the self-reported perfectionism scale (SOP) was 37.41 (SD 12.95), and the score obtained on the socially prescribed perfectionism scale (SPP) was 22.13 (SD 8.69). These values differed (*p* < 0.0001), indicating higher scores obtained in the SOP compared to the SPP.

Correlations between clinical perfectionism (SOP, SPP dimensions, and a total score obtained at CAPS) and EDI-3 scales were, in particular, the following: (1) over control (OC) and its sub dimensions perfectionism (P) and asceticism (A); (2) drive for thinness (DT correlated with SOP and total CAPS); (3) interceptive deficits (IDs); (4) interpersonal problems (correlated with SPP and total CAPS); (5) general psychological maladjustment composite (GPMC); (6) finally, higher perfectionism scores correspond to a greater depressive symptomatology in the CBCL scale (AFP). These were moderate correlations (>0.3 and <0.7) (Table 4).

Perfectionism did not correlate with the following clinical variables: duration of disease, BMI, and age, nor with the psychopathological dimensions investigated with the BUT questionnaire (Table 4).

## 4. Discussion

In this voxel-based morphometry (VBM) study on adolescent patients with AN-R, an attempt was made to address the need for greater methodological rigor to minimize the interference of confounding factors [33]. Morphometric alterations correlate with a lower body mass index (BMI), suggesting a pseudo-atrophic effect due to malnutrition. No macro-structural changes in the white matter (WM) were observed, indicating that WM is less susceptible to malnutrition-related alterations than gray matter (GM). The decrease in the global GM volume with a concomitant increase in CSF volume in the AN-R sample compared to the control group confirms a recurring finding in the literature [24], with evidence of a rather large global loss of the GM in the adolescent population, compared to adult samples [34,35]. The difference between the AN-R patients and controls assumes greater robustness, since it is independent from both age and total intracranial volume. This type of alteration is generally defined as ‘pseudo atrophy’, since it is a consequence of malnutrition on encephalic tissues, as also demonstrated on an ABA (Activity-Based Anorexia) mouse model of AN [36,37]. The absence of white matter (WM) alterations in our study is in line with a previous VBM investigation on 12 adolescents with AN-R (mean age: 14.5 years; mean BMI: 14.8 kg/m^2^) [38]. Weight reduction in the current sample was sufficient to cause a decrease in the GM volume, but not to produce noticeable volume alterations in WM, which needs more extreme BMI conditions to change its macrostructure and, according to some studies, when altered takes on a worse prognostic value [39]. This hypothesis was confirmed by a longitudinal study in which alterations in the GM and WM during the acute phase of the disease were followed by a restitutio ad integrum of the GM alone after weight recovery, indicating a certain WM resistance to recovery [39].

### 4.1. Regional Level Results: Cerebellum

In agreement with previous work on adolescents with AN-R [35,40,41], a decrease in gray matter volume, compared to the control group, was found at the cerebellar level. Volumetric reductions in cerebellar GM volume have been detected also by other research groups, especially in adolescent patients with AN, as pointed out in a review on this topic. This result suggests the pathophysiological implication of the cerebellum in AN. Indeed, the cerebellum is connected to the hypothalamus, an area in which a contextual decrease in GM volume has often been reported [34], and the cerebellum–hypothalamic circuits seem to be involved in the regulation of food intake and related to the sense of satiety [42]. Moreover, this finding has been further corroborated through resting state functional MRI studies, which have demonstrated the presence of altered intrinsic connectivity in the cerebellar vermis of AN patients [43]. It is long past time that the cerebellum has been recognized as having more complex roles than the historical and well-known functions of balance, learning, and motor coordination. Indeed, several investigations highlighted its role also in cognition and emotional experience [44,45,46], and there is evidence of cerebellar connections with areas in which we detected a decrease in volume, including the dorsolateral prefrontal cortex, medial frontal cortex, and parietal areas [47,48,49]. Moreover, some studies evidenced that cerebellar dysfunction may correlate with traits evident in individuals with AN, such as anxiety [50], ritual and stereotypical behaviors, as well as dysphoria, depression, and ruminative and obsessive thinking [45,51]. However, some aspects point to an involvement of the cerebellum secondary to the effects of malnutrition. Indeed, cerebellar maturation extends long into the adolescent period, making this structure more vulnerable to multiple environmental and nutritional factors [46]. An additional aspect supporting the cerebellar involvement secondary to malnutrition relies on the restitutio ad integrum cerebellar gray matter volume after weight recovery [39,41], with a correlation between volumes and BMI increases. Finally, increased cerebellar involvement, along with decreased white matter volume, has a predictive value relative to weight at a one-year follow-up [35]. In our sample, cerebellar volumes did not show any correlation with disease duration. Mixed findings exist on this topic; some studies on adults [52,53] reported a correlation between lower cerebellar volumes and longer disease duration, while a recent report did not confirm this result in a population of adolescents with acute AN [54]. An intriguing hypothesis is that cerebellar alteration derived from starvation may, in turn, become a factor in the maintenance of the AN disorder, reinforcing altered behavior patterns related to feeding and psychopathological traits [42].

### 4.2. Regional Differences at the Supratentorial Level

Statistically significant differences between AN patients and controls were also found in the volume decrease in frontal lobes bilaterally, although not entirely symmetrically. Again, this is a finding previously reported in the literature [9,11,55]. The involvement of the middle frontal gyrus bilaterally (MFG; Brodmann’s areas 8 and 9), the superior frontal gyrus (BA 6), the medial frontal gyrus (BA 8 on the left), and the precentral gyrus (BA 4 on the right) could suggest a link between these structures and the dysfunctions at the level of the cognitive patterns and executive functions (moreover, not without emotional and psychopathological repercussions) found in anorexic patients, and concerning, for example, the following: decision-making processes when set-shifting skills are required [56], due to the presence in these patients, of poor cognitive flexibility [57], or when the level of uncertainty increases, due to poor uncertainty tolerance, which is reflected in cognitive and emotional processes, expressing itself in harm avoidance behaviors, but also poor central coherence (focus on details losing the overall view of the big picture) [58], and other aspects of cognitive rigidity previously described [59].

From a pathophysiological point of view, dysfunctions in fronto-striatal circuits have been called into question [60]. Already more than 20 years ago, the importance of an alteration at the frontotemporal level was described, as opposed to the previous view of anorexia as a disorder related to the hypothalamic involvement [61]. There are also interesting theories, supported by fMRI studies [62], regarding the presence of an imbalance between bottom-up (midbrain) regions whose activation would be reduced, to the advantage of top-down hyper activation in the pre-orbitofrontal and the DLPFC [63], which would be reflected in greater cognitive control.

One region where a significant difference in GM was found bilaterally between cases and controls, in terms of volume reduction, is the MFG (Brodmann area 8 -BA8-). There is evidence for a role of this region in decision-making tasks, within broader neural networks [64]. Moreover, MFG appears to have different activation depending on the risk attitude of the subject examined [65]. In this framework, intolerance of uncertainty is a common trait and underlies perfectionism, harm avoidance, and the anxiety and obsessive symptoms found in eating disorders, which may be a maintenance factor in association with cognitive, interoceptive, and affective symptoms [66].

A volumetric decrease in the supplementary motor area (SMA; BA6) has been repeatedly observed in AN [9,24,43,67,68]. The SMA is primarily implicated in voluntary movement planning and control, as well as in the adaptation of the motor behavioral response based on contextual changes [56]. Therefore, it is plausible that an alteration of this region may contribute to the cognitive-behavioral inflexibility frequently reported in AN patients [57], supporting self-induced food restriction [24], also in line with the role of SMA in appetite suppression [69].

Finally, the superior frontal gyrus is involved in introspection [70], with evidence of reduced activation in AN patients while processing self-image [71]. In particular, fMRI studies emphasize reduced sensorimotor network (SMN) activity in the SMA of anorexic patients [72], reflecting the functional involvement of this area along with the primary motor area and sensorimotor cortex, in which we detected a reduced gray matter volume. An fMRI study demonstrated altered activation in AN patients during tasks that involved behavioral set-shifting in the SMN comprising BA 3, 4, and 6 [13], all regions in which the VBM analysis we conducted found a decreased GM volume. A recent meta-analysis on 21 studies highlighted a reduction in gray matter volume in three main areas in AN individual: the cingulate, frontal, and parietal cortex [24], while Titova et al. described volume alterations in VBM studies in the parietal region [73]. Alterations in the parietal cortex, the site of the somatosensory areas, may be implicated in the maintenance of alterations at the level of body representation [74]. Regarding the involvement of this area, there are studies oriented for its role in consciousness and of one’s own body, understood as something more than the simple summation of the proprioceptive, sensory, and interoceptive stimuli derived from it [75]. There is also evidence of altered activation patterns of the parietal cortex in AN patients when observing digitally distorted images of their own bodies [61], and it recently has been hypothesized that this area could be the basis of a nuclear symptom of AN [76], related to difficulties in visuospatial integration [77], which are, in turn, supported by the parietal regions [55,78]. In addition, a volumetric reduction in gray matter was found in the left middle frontal gyrus (BA 9). This area corresponds functionally to the dorsolateral prefrontal cortex (DLPFC), which is involved in cognitive functions such as reward evaluation, maintenance of attention and working memory, error recognition, motor planning, and cognitive and emotional control [21,79], also related to food aspects, as evidenced in studies in which the applications of DLPFC-targeted neuromodulation therapies (tDCS transcranial stimulation) implied a reduction in food intake and food craving in healthy, overweight individuals [80]. In this framework, higher levels of self-criticism in an AN patient are related to greater activity of the DLPFC region during a passive viewing of hostile compared to neutral stimuli [81].

### 4.3. Correlation Between Neuroanatomical Data and BMI

Previous studies have explored the relationship between brain structural volumetric abnormalities and BMI, and the results seem often contradictory. Five studies report a correlation between the BMI and a volumetric decrease in brain areas investigated by the VBM method [43,52,77,82,83], and more broadly, by MRI data [55,84], while other studies do not confirm this evidence [14,53,67,85,86].

### 4.4. Correlation Between Clinical Perfectionism and Other Psychopathological Dimensions

The statistically significant difference obtained in the scores of the two dimensions of clinical perfectionism between the higher scores obtained at the SOP (self-oriented perfectionism) compared to those reported at the SPP (socially prescribed perfectionism) aligns with the results of studies related to the dimensions of perfectionism, according to which the SOP is more representative of the population of anorexic patients than the SPP [87,88].

Clinical perfectionism also correlates with some EDI-3 scales. In particular, correlation with the Hypercontrol (OC) domain and its sub dimensions, perfectionism (P) and asceticism (A), confirms a good validity of the scales, which assess constructs that are partly overlapping (CAPS and perfectionism subscale of the EDI-3) and closely interrelated (dimensions of perfectionism and asceticism). As for the drive for thinness (DT), a specific nuclear symptom of eating disorders, this domain correlates with the SOP subscale, underscoring how the pursuit of an ideal of thinness rests on rigid self-imposed patterns [89]. The correlation with interoceptive deficits (IDs) reflects the imbalance between the top-down frontal control structures, hyperactive in AN and correlated with the perfectionism dimension, against the impaired functioning of the ventral, bottom-up structures, to which the interoceptive awareness dimension (one above all the insula) is generally referred, which is impaired in these patients [3].

Interpersonal problems (IPPs), in addition to the total CAPS score, correlate with SPP, testifying to how the perception of the other as judgmental is not limited to an internalizing dimension, but rather is extrinsic at the interpersonal level, with a possible deterioration of the relationships.

The fact that higher levels of clinical perfectionism are related to a higher degree of General Psychopathological Maladjustment on the EDI-3 and higher depressive symptomatology on the DSM-oriented “Affective Problems” scale of the CBCL suggests that this trait typical of anorexic pathology expands across various domains, to the point of burdening internalizing symptomatology and the overall perception of discomfort.

These findings allow the construct of perfectionism to be identified as a potential therapeutic target [90,91], linked to many other psychopathological aspects, as well as to dysfunctional cognitive styles, and thus to be read as a maintenance factor of anorexic pathology.

### 4.5. Limitations of the Study

One of the limitations of this work is the lack of data regarding BMI in the control group as well as the lateralization and quantification of cognitive abilities in the two groups. Moreover, it was not possible to administer the clinical questionnaires to the control group.

The clinical sample displayed a short disease duration on average (≤2 years in 83% of the sample), but included a few outliers, an aspect that could prove confounding from a statistical point of view. Participants belong to a hospitalized clinical population, with a particularly severe picture in terms of physical and/or psychopathological conditions, the presence of psychiatric comorbidity, and the requirement of drug therapy. These aspects, in addition to representing confounding factors in the evaluation of brain volumes, do not allow to extend results to less impaired AN-R patients.

On the other hand, some strengths of the study must be recognized. Specifically, we can benefit from a quite large sample of 47 adolescent patients, which are homogeneous in terms of gender (all females) and pathology type (restrictive subtype of anorexia nervosa). The mean age of onset found in our sample is 13.7 years (DS 1.5), ranging from 10.3 to 16.6 years. Recent studies attest to the existence of a peak onset of restrictive anorexia nervosa around 15 years of age; however, earlier onset is on the rise. In this sense, we can assert that our sample is representative of the current European situation [92].

In the present study, no patient was in a state of acute malnutrition/dehydration due, for example, to a condition of fasting prior to the acquisition of MRI images, since only patients adhering to dietary plans agreed upon with the clinical team were selected.

Although no a priori power analysis was conducted, a post hoc power calculation based on significant differences between groups in global gray matter volume (Cohen’s d = 1.32) confirmed that the sample we used (n = 86) provides statistical power > 0.99 to detect the effects between groups at α = 0.05. This confirms the robustness of our results despite the inherent limitations of clinical recruitment in rare adolescent populations.

## 5. Conclusions

In conclusion, AN-R patients displayed a global involvement of gray matter (GM) decrease with a concomitant CSF increase that correlates with a lower body mass index (BMI), suggesting a likely pseudo-atrophy of the brain. The failure to find macro structural changes in white matter (WM) confirms the lower susceptibility of the WM to starvation. However, the presence of microstructural changes in white matter, which can be investigated by more specific neuroimaging techniques, such as DTI, cannot be excluded. Decreases in gray matter volume at the regional level (cerebellum, frontal, and parietal regions) affect areas of significance in the pathophysiology of AN-R, and are correlated with the lower BMI of the patients.

## Figures and Tables

**Table 1:** Demographic and clinical characteristics of the AN-R study population.

AN-R Cases (n)	Mean Age ± SD (Years)	Mean BMI ± SD (kg/m^2^)	Mean Age of Onset ± SD (Years)	Mean Disease Duration ± SD (Months)	Hyperactivity (%)	Amenorrhea (%)	Familiarity (n)
47	15.0 ± 1.4	15.3 ± 1.3	13.7 ±1.5	17.0 ± 9.6	55	72 Secondary 13 Primaries	Absent: 11 Positive for ED: 21 Psychiatric: 15

**Table 2:** Demographic data and global volume comparisons.

Variables	ALL (Mean)	ALL (SD)	AN-R (Mean)	AN-R (SD)	CTRL (Mean)	CTRL (SD)	*p*-Value
Age (months)	182	25	180	21	184	29	0.48
TIV (l)	1.31	0.09	1.31	0.11	1.31	0.08	0.77
GM (l)	0.72	0.06	0.69	0.06	0.76	0.04	1 × 10^−6^
WM (l)	0.37	0.03	0.37	0.03	0.38	0.03	0.86
CSF (l)	0.21	0.07	0.24	0.07	0.17	0.04	3 × 10^−7^

**Table 3:** VBM analysis with the application of a 500-voxel threshold, and with a value of *p* < 0.05. Seven major regions are identified, which are further expanded into their main sub clusters. The Montreal Neurological Institute (MNI) and the Talairach (Tal) coordinates of these regions are provided, followed by their description at the level of the hemispheres, lobes, and their sub regions. The correspondence to the Brodmann areas involved is also reported. Legend: Right Cerebrum, RC; Left Cerebrum, LC; Right Cerebellum, RCL; Left Cerebellum, LCL; Brodmann Area (BA). *, Not detected.

Record Number	x_MNI_	y_MNI_	z_MNI_	x_Tal_	y_Tal_	z_Tal_	Hemisphere	Lobe	Gyrus	Brodmann Area
**1**	**34**	**−24**	**52**	**30**	**−28**	**49**	**RC**	**Frontal Lobe**	**Precentral Gyrus**	**BA 4**
	39	−16	42	34	−20	40	RC	Frontal Lobe	Precentral Gyrus	BA 4
	51	−9	46	45	−14	45	RC	Frontal Lobe	Precentral Gyrus	BA 4
**2**	**27**	**38**	**36**	**23**	**29**	**39**	**RC**	**Frontal Lobe**	**Middle Frontal Gyrus**	**BA 8**
	24	36	45	20	27	47	RC	Frontal Lobe	Superior Frontal Gyrus	BA 8
	12	34	46	9	26	48	RC	Frontal Lobe	Medial Frontal Gyrus	BA 8
**3**	**−46**	**−12**	**52**	**−44**	**−17**	**47**	**LC**	**Parietal Lobe**	**Postcentral Gyrus**	**BA 3**
	−42	−20	38	−40	−22	35	LC	Parietal Lobe	Postcentral Gyrus	BA 3
	−54	−9	44	−51	−13	41	LC	Frontal Lobe	Precentral Gyrus	BA 4
**4**	**−15**	**−74**	**−15**	**−14**	**−68**	**−16**	**LCL**	**Posterior Lobe**	**Declive**	*
	36	−46	−40	32	−41	−35	RCL	Posterior Lobe	Cerebellar Tonsil	*
	28	−40	−44	25	−35	−37	RCL	Posterior Lobe	Cerebellar Tonsil	*
**5**	**−50**	**28**	**26**	**−47**	**22**	**28**	**LC**	**Frontal Lobe**	**Middle Frontal Gyrus**	**BA 9**
	−45	38	12	−42	32	17	LC	Frontal Lobe	Middle Frontal Gyrus	BA 46
	−42	28	28	−40	22	31	LC	Frontal Lobe	Middle Frontal Gyrus	BA 9
**6**	**−2**	**36**	**45**	**−2**	**27**	**47**	**LC**	**Frontal Lobe**	**Medial Frontal Gyrus**	**BA 8**
	−9	28	40	−9	21	42	LC	Limbic Lobe	Cingulate Gyrus	BA 32
	2	27	50	−0.1	19	50	LC	Frontal Lobe	Superior Frontal Gyrus	BA 8
**7**	**0**	**15**	**60**	**−1**	**6**	**58**	**LC**	**Frontal Lobe**	**Superior Frontal Gyrus**	**BA 6**
	9	−16	44	6	−20	41	RC	Frontal Lobe	Paracentral Lobule	BA 31
	0	−6	48	−1	−11	46	LC	Frontal Lobe	Paracentral Lobule	BA 31

**Table 4:** Values of correlation and corresponding *p* values between regions with decreased GM and clinical and psychopathological dimensions. A significant correlation is detected only between the altered GM volumes in AN-R and the BMI.

Variable	Correlation Coefficient	*p*-Value
BMI	0.45	<0.01
Disease duration	−0.05	0.78
SOP Self-Oriented Perfectionism, CAPS	−0.00	0.98
SPP Socially Prescribed Perfectionism, CAPS	−0.17	0.92
DT Drive for Thinness, EDI-3	0.10	0.53
BD Body Dissatisfaction, EDI-3	0.22	0.18
ERDC Eating Disorder Risk, EDI-3	0.14	0.38
IC Inadequacy, EDI-3	0.10	0.52
IPC Interpersonal Problems, EDI-3	−0.08	0.63
APC Affective Problems, EDI-3	−0.19	0.25
OC Hyper-Control, EDI-3	0.02	0.88
GPMC General Psychological Maladjustment Composite, EDI-3	−0.11	0.47
GSI Global Severity Index, BUT	0.08	0.64
WP Weight Phobia, BUT	0.07	0.66
BIC Body Image Concerns, BUT	−0.06	0.70
CSM Compulsive Self-Monitoring, BUT	0.13	0.41
AFP Affective Problems, CBCL	−0.08	0.61

## Data Availability

Data are unavailable due to privacy or ethical restrictions.

## Supplementary Materials

The following supporting information can be downloaded at: https://www.mdpi.com/article/10.3390/nu17162620/s1, Table S1: Clusters of altered gray matter regions derived from the voxel-based comparison between participants with anorexia nervosa (AN-R) and healthy controls (CTRLs) in terms of Region of Interest cerebral gray matter volumes (mL). TIV weighted MRI measures. All the results shown in the table are significant (*p* < 0.05). The rows are sorted from the lowest *p* to the highest *p*. Until record 88, the *p* is less than 0.01.
