# Supplementary material for: Body Mass Index Impacts on Gray Matter Volume in Developmental Restrictive Anorexia Nervosa: A Voxel-Based Morphometry Study
Source: Nutrients. 2025 Aug 13;17(16):2620. doi: 10.3390/nu17162620 (PMC12389083; doi:10.3390/nu17162620)
Supplement: Supplementary file 1 [file nutrients-17-02620-s001.zip › nutrients-3767521-supplementary.pdf]

nutrients

MDPI

Supplementary materials

Table S1. Clusters of altered grey matter regions derived from the voxel-based comparison between participants with anorexia nervosa (AN-R) and healthy controls (CTRL) in terms of Region of Interest cerebral gray matter volumes (ml). TIV weighted MRI measures. All the results shown in the table are significant (p<0.01). The rows are sorted from the lowest p to the highest p. Legend: Right Cerebrum, RC; Left Cerebrum, LC; Right Cerebellum, RCL; Left Cerebellum, RCL; Brodmann Area (BA).

Record Number	Hemisphere	Lobe	Gyrus	Brain Area	Range (mm)
**1**	**RC**	**Frontal Lobe**	**Superior Frontal Gyrus**	**BA 8**	**1**
2	RC	Frontal Lobe	Middle Frontal Gyrus	BA 8	0
3	RC	Frontal Lobe	Medial Frontal Gyrus	BA 8	2
**4**	**RC**	**Frontal Lobe**	**Precentral Gyrus**	**BA 4**	**0**
5	RC	Parietal Lobe	Postcentral Gyrus	BA 3	3
6	RC	Frontal Lobe	Precentral Gyrus	BA 4	0
**7**	**RCL**	**Posterior Lobe**	**Cerebellar Tonsil**	*	**0**
8	LCL	Posterior Lobe	Declive	*	0
9	RCL	Anterior Lobe	Culmen	*	0
**10**	**RC**	**Sub-lobar**	**Insula**	**BA 13**	**1**
11	RC	Sub-lobar	Insula	BA 13	1
12	RC	Temporal Lobe	Superior Temporal Gyrus	BA 22	0
**13**	**LCL**	**Posterior Lobe**	**Cerebellar Tonsil**	*	**0**
14	LCL	Anterior Lobe	Culmen	*	0
**15**	**LC**	**Parietal Lobe**	**Postcentral Gyrus**	**BA 3**	**1**
16	LC	Parietal Lobe	Postcentral Gyrus	BA 3	3
17	LC	Frontal Lobe	Precentral Gyrus	BA 4	0
**18**	**LC**	**Frontal Lobe**	**Medial Frontal Gyrus**	**BA 6**	**0**
19	LC	Frontal Lobe	Medial Frontal Gyrus	BA 8	0
20	LC	Limbic Lobe	Cingulate Gyrus	BA 32	0
**21**	**LC**	**Frontal Lobe**	**Middle Frontal Gyrus**	**BA 9**	**2**
22	LC	Frontal Lobe	Middle Frontal Gyrus	BA 8	0
23	LC	Frontal Lobe	Superior Frontal Gyrus	BA 8	0
**24**	**RC**	**Parietal Lobe**	**Inferior Parietal Lobule**	**BA 40**	**0**
**25**	**RC**	**Temporal Lobe**	**Middle Temporal Gyrus**	**BA 39**	**1**

26	RC	Temporal Lobe	Middle Temporal Gyrus	BA 39	0
27	RC	Temporal Lobe	Middle Temporal Gyrus	BA 37	1
**28**	**RC**	**Occipital Lobe**	**Praecuneus**	**BA 31**	**0**
29	RC	Occipital Lobe	Cuneus	BA 7	1
30	LC	Occipital Lobe	Praecuneus	BA 23	1
**31**	**RC**	**Parietal Lobe**	**Praecuneus**	**BA 7**	**1**
32	RC	Parietal Lobe	Praecuneus	BA 7	0
33	LC	Parietal Lobe	Praecuneus	BA 7	0
**34**	**RC**	**Temporal Lobe**	**Middle Temporal Gyrus**	**BA 37**	**2**
35	RC	Temporal Lobe	Fusiform Gyrus	BA 37	1
**36**	**LC**	**Sub-lobar**	**Caudate**	**Caudate Head**	**0**
37	LC	Limbic Lobe	Anterior Cingulate	BA 25	0
38	LC	Limbic Lobe	Anterior Cingulate	BA 24	2
**39**	**LC**	**Frontal Lobe**	**Medial Frontal Gyrus**	**BA 6**	**2**
40	RC	Frontal Lobe	Paracentral Lobule	BA 31	0
41	RC	Frontal Lobe	Paracentral Lobule	BA 31	2
**42**	**LC**	**Sub-lobar**	**Insula**	**BA 13**	**1**
43	LC	Sub-lobar	Insula	BA 13	0
**44**	**LC**	**Parietal Lobe**	**Angular Gyrus**	**BA 39**	**1**
45	LC	Parietal Lobe	Inferior Parietal Lobule	BA 7	0
46	LC	Parietal Lobe	Inferior Parietal Lobule	BA 40	2
**47**	**LC**	**Frontal Lobe**	**Precentral Gyrus**	**BA 6**	**1**
48	LC	Sub-lobar	Insula	BA 13	1
49	LC	Temporal Lobe	Superior Temporal Gyrus	BA 22	0
**50**	**LC**	**Frontal Lobe**	**Medial Frontal Gyrus**	**BA 10**	**0**
**51**	**LC**	**Frontal Lobe**	**Inferior Frontal Gyrus**	**BA 13**	**4**
52	LC	Frontal Lobe	Inferior Frontal Gyrus	BA 47	3
53	LC	Frontal Lobe	Inferior Frontal Gyrus	BA 45	4
**54**	**RC**	**Sub-lobar**	**Insula**	**BA 13**	**2**
55	RC	Temporal Lobe	Superior Temporal Gyrus	BA 13	2
56	RC	Temporal Lobe	Superior Temporal Gyrus	BA 22	0
**57**	**LC**	**Temporal Lobe**	**Middle Temporal Gyrus**	**BA 21**	**0**
58	LC	Temporal Lobe	Middle Temporal Gyrus	BA 21	1
**59**	**RC**	**Sub-lobar**	**Insula**	**BA 13**	**0**

**60**	**LC**	**Temporal Lobe**	**Fusiform Gyrus**	**BA 37**	**1**
**61**	**RC**	**Sub-lobar**	**Thalamus**	**Medial Dorsal Nucleus**	**0**
**62**	**LC**	**Frontal Lobe**	**Precentral Gyrus**	**BA 6**	**0**
63	LC	Frontal Lobe	Precentral Gyrus	BA 6	0
**64**	**RC**	**Temporal Lobe**	**Middle Temporal Gyrus**	**BA 39**	**3**
**65**	**RC**	**Limbic Lobe**	**Para hippocampal Gyrus**	**BA 27**	**1**
**66**	**RC**	**Limbic Lobe**	**Cingulate Gyrus**	**BA 24**	**0**
**67**	**RC**	**Limbic Lobe**	**Anterior Cingulate**	**BA 32**	**4**
**68**	**RC**	**Parietal Lobe**	**Supramarginal Gyrus**	**BA 40**	**3**
69	RC	Parietal Lobe	Supramarginal Gyrus	BA 40	4
70	RC	Parietal Lobe	Supramarginal Gyrus	BA 40	5
**71**	**RC**	**Parietal Lobe**	**Praecuneus**	**BA 31**	**0**
72	RC	Limbic Lobe	Cingulate Gyrus	BA 31	1
73	LC	Parietal Lobe	Praecuneus	BA 31	2
**74**	**LC**	**Frontal Lobe**	**Inferior Frontal Gyrus**	**BA 47**	**2**
**75**	**RC**	**Temporal Lobe**	**Fusiform Gyrus**	**BA 37**	**0**
**76**	**RC**	**Parietal Lobe**	**Inferior Parietal Lobule**	**BA 40**	**1**
**77**	**LC**	**Parietal Lobe**	**Superior Parietal Lobule**	**BA 7**	**1**
**78**	**LC**	**Temporal Lobe**	**Superior Temporal Gyrus**	**BA 22**	**0**
**79**	**RC**	**Temporal Lobe**	**Superior Temporal Gyrus**	**BA 22**	**1**
80	RC	Temporal Lobe	Superior Temporal Gyrus	BA 22	2
**81**	**LC**	**Frontal Lobe**	**Superior Frontal Gyrus**	**BA 9**	**1**
**82**	**LC**	**Frontal Lobe**	**Medial Frontal Gyrus**	**BA 6**	**2**
**83**	**LC**	**Parietal Lobe**	**Praecuneus**	**BA 7**	**2**
**84**	**LC**	**Temporal Lobe**	**Superior Temporal Gyrus**	**BA 42**	**1**
**85**	**RC**	**Temporal Lobe**	**Superior Temporal Gyrus**	**BA 38**	**0**
**86**	**LC**	**Frontal Lobe**	**Medial Frontal Gyrus**	**BA 9**	**0**
**87**	**LC**	**Frontal Lobe**	**Middle Frontal Gyrus**	**BA 10**	**5**
**88**	**Right Brainstem**	**Midbrain**	*****	**Mammillary Body**	**1**
